# Does influenza pandemic preparedness and mitigation require gain‐of‐function research?

**DOI:** 10.1111/irv.12458

**Published:** 2017-06-24

**Authors:** Dillon C. Adam, Daniel Magee, Chau M. Bui, Matthew Scotch, C. Raina MacIntyre

**Affiliations:** ^1^ School of Public Health and Community MedicineUNSWSydneyNSWAustralia; ^2^ Biodesign Center for Environmental SecurityBiodesign InstituteArizona State UniversityTempeAZUSA; ^3^ Department of Biomedical InformaticsCollege of Health SolutionsArizona State UniversityTempeAZUSA; ^4^ College of Public Service & Community SolutionsArizona State UniversityTempeAZUSA

**Keywords:** influenza, pandemics, public health surveillance

## Abstract

The risk and benefits of gain‐of‐function studies on influenza A have been widely debated since 2012 when the methods to create two respiratory transmissible H5N1 mutant isolates were published. Opponents of gain‐of‐function studies argue the biosecurity risk is unacceptable, while proponents cite potential uses for pandemic surveillance, preparedness and mitigation. In this commentary, we provide an overview of the background and applications of gain‐of‐function research and argue that the anticipated benefits have yet to materialize while the significant risks remain.

## 1 INTRODUCTION

Within the last century, humanity has faced three devastating human influenza pandemics: H1N1 in 1918, H2N2 in 1957 and H3N2 in 1968.^1^ Collectively, these three pandemics are estimated to have resulted in approximately 46 million deaths and over 500 million infections.^2^ In recent decades, the rate of emergence of zoonotic influenza A serotypes in humans has increased, supporting the consensus that a future influenza pandemic of zoonotic origin is on the horizon.^3^, ^4^, ^5^ In 1996, a novel serotype, H5N1, was isolated from a goose in Hong Kong with the first human cases (n=18) being recorded following exposure to poultry in 1997.^6^ Since official World Health Organization (WHO) reporting began in 2003, 859 human cases and 453 deaths have been recorded due to sporadic zoonotic transmission of H5N1 from avian species.^7^ However, small clusters of un‐sustained human‐to‐human transmission have been documented in rural areas with low population density.^8^, ^9^, ^10^, ^11^ As a highly pathogenic avian influenza (HPAI) virus with unprecedented endemicity in birds and high mortality, many have postulated that H5N1 may be the source of the next human influenza pandemic.^12^, ^13^, ^14^

Unlike past pandemics, the ease of international travel in the modern age means that viruses can spread around the globe in a very rapid timeframe^15^, ^16^; the 2009 pandemic of swine‐origin H1N1(H1N1pdm09) is a testament to the ongoing risk of emerging infections in a globalized world. While the total burden of H1N1pdm09 was similar to a severe seasonal influenza epidemic, advances in medicine such as extra‐corporeal membrane oxygenation and antibiotics for secondary bacterial infection have vastly improved survival compared to 1918 making relative pandemic predictions of disease burden difficult.^17^, ^18^ However, modelling studies have shown that a highly contagious H5N1 virus could infect up to 50% of the population globally^19^: 3.57 billion people as of 2013.^20^ Recognizing this increasing vulnerability, significant work has been conducted to‐date in an attempt to identify influenza lineages at high risk of pandemic emergence. It is widely accepted that the pandemic potential of H5N1 is largely dependent on mutations that enable sustainable human‐to‐human transmission,^21^ and gain‐of‐function studies have offered a novel means to identify the relationship between mutant genotypes and potentially transmissible phenotypes. Using H5N1 as a case study, we review the role of gain‐of‐function (GOF) research as a tool for surveillance of pre‐pandemic influenza A. We also consider alternative approaches for pre‐pandemic influenza surveillance as well as the acceptability and risk of GOF research, which has been debated extensively in the past,^22^, ^23^, ^24^, ^25^, ^26^, ^27^, ^28^ from a surveillance and preparedness context.

## 2 HEMAGGLUTININ RECEPTOR SPECIFICITY—A CRITICAL RISK FACTOR FOR PANDEMIC EMERGENCE

It is understood that the host range of avian influenza viruses is partially determined by the binding specificity of the hemagglutinin (HA) protein on the virus’ surface to either human or avian cell receptors.^29^, ^30^, ^31^, ^32^, ^33^ Most functional interactions between surface cell receptors and influenza A viruses occur via the receptor binding site (RBS) of HA: a structural domain formed by the 190‐helix, 220‐loop and the 130‐loop. For human respiratory colonization, it is necessary that mutations alter the specificity from avian α2,3‐linked sialic acid (α2,3‐SA) receptors found in the avian intestinal tract,^34^, ^35^ to mammalian α2,6‐linked (α2,6‐SA) receptors located on the epithelial cells of the human upper respiratory tract.^36^, ^37^, ^38^, ^39^, ^40^, ^41^ Crystal structure studies have revealed a widening of the RBS in mammalian‐adapted HA to facilitate binding to the larger α2,6‐SA *cis*‐linked receptors.^42^, ^43^

HA receptor specificity is considered the third‐most important predictor of pandemic emergence following (i) human infection and (ii) airborne transmission in animal models according to the Centers for Disease Control and Prevention's (CDC) Influenza Risk Assessment Tool (IRAT), which also incorporates the results of GOF research.^44^ Other viral factors such as improved polymerase efficiency and HA pH activation are similarly necessary for efficient human‐to‐human transmission, but are not alone sufficient for colonization and therefore considered less significant. Therefore, focusing research efforts towards potentially pandemic H5‐HA mutations appears justified.^45^, ^46^

## 3 GOF RESEARCH—THE RELATIONSHIP BETWEEN TRANSMISSION GENOTYPE AND PHENOTYPE

In 2012, two groups successfully demonstrated a H5N1 transmissible phenotype in ferrets, the best surrogate animal model for human influenza research.^47^, ^48^ Using reverse genetics, they identified mutant genotypes responsible for mammalian adaptation. Many of these mutations have been previously shown to increase the binding of HA to mammalian α2,6‐SA receptors albeit in isolation and without demonstrated ferret transmission.^49^, ^50^, ^51^, ^52^ These mutant genotypes have been partially identified in natural H5N1 variants currently circulating in avian species and cases of human H5N1 infection today.^53^, ^54^ Numerous GOF studies have identified additional mutants of pandemic concern, a summary of which is aggregated by the CDC^55^ and others.^21^ Translation of these mutants of concern into predictors of pandemic emergence has since been upheld as an informative tool for current public health surveillance and preparedness efforts: to‐date, the results of GOF research have been used to inform pandemic influenza surveillance activities as well as efforts in pandemic preparedness planning and response in parts of Asia and the Middle East.^56^, ^57^

## 4 APPLICATIONS, LIMITATIONS AND RISK IN GOF RESEARCH

Following an outbreak of H5N1 in Cambodia in 2013 that totalled 26 human cases and 14 deaths,^58^ the CDC rapidly deployed a response team to conduct control measures and epidemiological investigations. Here it was determined that many cases had become infected with strains naturally possessing GOF genotypes partially matched to the laboratory transmissible strain.^59^ Following the initial CDC investigation, the WHO coordinated the development of a candidate vaccine. However, as the roll out of a matched vaccine is 3‐6 months at a minimum using current technology in embryonated eggs,^60^ there could not have been enough time to materially impact the epidemic peak. Other mitigation measures such as antivirals, personal protective equipment (PPE) and non‐pharmaceutical interventions are more critical in the early pandemic phase. However, the rapid response in Cambodia is thought to have reduced the possible time‐to‐market by at least a month notwithstanding the impact of immediate control measures employed.^56^ It was later determined that no human‐to‐human transmission had occurred, and that the mutant strains had arisen following human infection rather than a precursor virus in poultry. Yet at the time, the precautionary principle was invoked to prevent a potential pandemic. Whether the intervention prevented a pandemic, however, cannot be proven. Beyond this specific case, the results of H5N1 GOF studies have been supported as one method to improve our understanding of other avian influenza viruses in the wild such as H7N9, considering many of the experimental GOF mutants identified are currently circulating in nature.^53^, ^54^

However, the predictive value of GOF mutants is not without its limitations. Due to the variable effect of mutations in strains with different genetic backgrounds, mutations in one strain may not confer the same transmissible phenotype in another. Studies have demonstrated that when applied to more recent strains, such as A/Chicken/Vietnam/093/2008, the same ferret transmissible genotype experimentally determined in vivo using A/Vietnam/1194/2004 and A/Indonesia/5/2005 did not confer the transmissible phenotype previously demonstrated.^61^ This evidence undermines the value and generalizability of molecular surveillance activities based on the results of GOF studies to currently circulating strains.

Gain‐of‐function research also poses significant risk to the greater global community as dual‐use research of concern (DURC), that is, research that is intended for good, but either through accidental or intentional misuse can cause significant harm to human health. Opponents of GOF research argue the threat of potential laboratory accidents sparking an unnatural pandemic or bioterrorism arising from the publication and replication of GOF methodologies is unacceptable. Specifically, GOF research cannot materially impact epidemic control such as through the development of a pre‐pandemic vaccine,^62^ nor does it increase the certainty of risk estimates for molecular surveillance purposes in which it has been proposed as shown above. A further point is the difficulty many experts have in distinguishing between natural vs unnatural disease events meaning accidental or deliberate outbreaks may go unrecognized.^60^ Some suggestions to mitigate the risk of GOF research have included limiting the number of laboratories allowed to do GOF research while increasing government oversight^63^; however, the European Union^64^ and the United States^65^ have both conducted risk‐benefit analyses on GOF research yet neither have been conclusive on the matter.

## 5 CONCLUSION

In conclusion, the net‐benefit argument supporting GOF research can be considered unjustified because the utility of GOF studies as a tool for pandemic risk assessment and surveillance activities is uncertain, while the overwhelming health risks to the greater global community due to the threat of unnatural pandemics and bioterrorism remain. Better risk assessment needs to be done. GOF research has proven useful in its purely scientific achievement of identifying the relationship between genotype and phenotype in vivo. Yet the limited generalizability of GOF research to the surveillance of currently circulating strains supports the need for further research into universal predictors of pandemic emergence. Alternative approaches to pandemic risk modelling have been proposed which are worth exploring, such as identifying molecular determinants of HA evolution.^66^ For example, studies have shown the protein structure of avian HA partially determines the mutation rate within the RBS and thus, greater opportunities for selection towards human respiratory cells, increasing risk.^67^, ^68^ Additionally, investing in greater diagnostic capacity to support surveillance systems, improving response plans for non‐pharmaceutical interventions and stockpiling antivirals and PPE are equally important during both the pre‐ and early pandemic phases.^69^ Our inability to accurately predict which subtype will emerge as the next pandemic demonstrates the need to research methods that are generalizable to other more recent emerging avian influenza A viruses such as H7N9 and H5N6 to which have high human exposure. Such methods could potentially increase the accuracy and certainty of pandemic risk estimates and more effectively direct surveillance preparedness activities to prevent and manage the next pandemic.

## AUTHOR CONTRIBUTIONS

DA contributed to the original draft preparation of the manuscript. DM and CB equally contributed to writing, review and editing of the manuscript. MS and CM contributed as senior authors, to funding acquisition, conceptualization, supervision, writing, review and editing of the manuscript.
